# Physical performance in elite male soccer under extreme heat: A case study of the 2025 FIFA Club World Cup

**DOI:** 10.1080/23328940.2026.2623745

**Published:** 2026-02-08

**Authors:** Adriano A. L. Carmo, Roberto C. S. Souza-Junior, Pedro H. S. Ferretti, Letícia A. Gontijo, Luciano S. Prado, Francisco Teixeira-Coelho, Thales N. Prímola-Gomes, Toby Mündel, Daniel P. Bitencourt, Rafael A. Torres-Pinto, Samuel P. Wanner

**Affiliations:** a Exercise Physiology Laboratory, School of Physical Education, Physiotherapy, and Occupational Therapy, Universidade Federal de Minas Gerais, Belo Horizonte, Brazil; b Department of Sport Sciences, Institute of Health Sciences, Universidade Federal do Triângulo Mineiro, Uberaba, Brazil; c Human Performance Laboratory, Department of Physical Education, Universidade Federal de Viçosa, Viçosa, Brazil; d Hydration Exercise and Temperature Laboratory, Department of Kinesiology, Brock University, St. Catharines, ON, Canada; e Jorge Duprat Figueiredo Foundation for Occupational Safety and Medicine, Florianópolis, Brazil

**Keywords:** Athletic performance, climate, extreme heat, global warming, heat illness, hot temperature, humidity, running, soccer, sports

## Abstract

The 2025 FIFA Club World Cup was held primarily during the summer season in the Northern Hemisphere, with reports of athletes exposed to significant environmental heat stress. We investigated whether environmental conditions, along with other factors (*e.g*., time of day, players’ age and field position, and club geographic origin), influenced physical performance in this tournament. Information about the performance during 57 matches (*n* = 1070 observations) was extracted from FIFA technical reports, whereas environmental conditions were obtained through mathematical modeling (ERA5 reanalysis). Linear mixed models were used to identify factors that explained variance in total distance covered and in distances covered at high, moderate, and low speeds. Mean wet-bulb globe temperature (WBGT) exceeded 28°C in 31 of the 57 matches analyzed, confirming that players were exposed to conditions of extreme heat illness risk. WBGT and air temperature explained total distance and distances at different speeds, while relative humidity explained distance only at high speeds (*p* < 0.001). More specifically, high WBGT, air temperature, and relative humidity values reduced the distances covered. Other factors also influenced players’ performance, including their position and age, time of day, and club geographic origin: longer distances were observed in midfielders/forwards, younger players, in the evening, and in clubs from cold climates (*p* < 0.05). In conclusion, the findings from this tournament, which featured many matches under extreme heat, highlight the multifaceted regulation of physical performance in soccer and emphasize the prominent role of environmental conditions in determining the distance players cover at different speeds.

## Introduction

Climate change, including global warming, has been accelerated by anthropogenic activity in the past decades, affecting different aspects of our lives, such as work productivity [1], participation in outdoor physical activities for recreation and conditioning [2,3], and the health of vulnerable individuals [4]. Climate change leads, among other undesirable consequences, to more frequent, prolonged, and intense heat waves [5,6]. Altogether, these changes increase athletes’ exposure to adverse environmental conditions during training and competition [7]. In this sense, environmental heat stress has become a concern for organizers of major sports events, coaching staff, and athletes, including those involved in the last two Summer Olympic Games [8,9].

Elite male soccer matches are contested at vigorous intensities, with an estimated metabolic rate of ~ 11 W/kg or 770 W for an athlete weighing 70 kg [10]. During an entire official soccer match, world-class defensive and central midfielders can run ~11.5 km, with some players running more than 13.0 km [11]. Depending on their positional role, players cover 9.7 – 10.1% of this total distance at speeds ≥20.0 km/h, which are classified as high-intensity activities [11]. Due to the vigorous intensity of their physical demands, soccer players generate considerable metabolic heat that must be dissipated to the environment to prevent overheating and its unwanted consequences on performance. Elevated body core temperature alters electroencephalographic activity, leading to increased perceived exertion and reduced voluntary activation of skeletal muscles [12]. Elevated skin temperatures, in turn, increase the sensation of heat [13] and narrow the core-to-skin temperature gradient, thereby requiring greater skin blood flow for effective heat loss [14]. In addition, body heat loss involves sweating, which may induce dehydration, reduced stroke volume, and increased heart rate [15]. Thus, thermoregulatory, cardiovascular, and perceptual strain act in an integrated manner to regulate performance, especially under environmental heat stress, when body heat loss is restricted [16].

As previously reviewed, environmental conditions influence the physical performance of soccer athletes [17]. A seminal study investigating the effect of environmental heat stress on soccer was conducted by Mohr et al. [18], who showed that elite male football players from two Scandinavian countries ran 26% less at higher speeds, covered a 7% shorter total distance, and had muscle and core temperatures ~1°C higher during an experimental match at an air temperature (T_a_) of 43°C than at 21°C. This finding was reproduced in subsequent studies [19–22]. For example, Nassis et al. [19] observed that environmental heat stress, as indicated by higher wet-bulb globe temperature (WBGT) values, was associated with shorter distances covered at high intensities during the 2014 FIFA World Cup matches. Most recently, Schwartz et al. [20] analyzed data from 1610 official matches in four professional soccer leagues (*i.e*., German Bundesliga 1 and 2, Japanese J-League, and Turkish SüperLig) and showed that total distance, high-speed and sprint distances, and number of sprints were all lower when WBGT or T_a_ was higher. Collectively, these findings stress the importance of environmental conditions for high-intensity running, a defining feature of soccer matches [23].

Most studies to date have investigated the effects of T_a_ or WBGT on the performance of soccer athletes [18–22,24]. While T_a_ is the most well-known environmental condition among the general population, WBGT is an index that considers natural wet-bulb and black globe temperatures in addition to T_a_, thereby accounting for the contributions of relative humidity, solar radiation, and wind speed to environmental heat stress. However, these studies did not address the independent role of the latter three environmental factors in determining athletic performance. Indeed, previous laboratory studies indicated that high relative humidity [25] or artificial radiation [26], when combined with elevated T_a_s, decreased prolonged performance during indoor exercise. Similar performance impairments were reported for the combination of still air and high T_a_ compared to faster air velocities [27].

In addition to environmental conditions, time of day also influences athletic performance, including preliminary evidence that soccer players ran greater distances at moderate speeds at night than in the afternoon [28]. Of note, soccer and other sports events are scheduled at different times throughout the day to serve the interests of television and streaming broadcasters. For example, to increase the worldwide audience for the 2025 FIFA Club World Cup matches, several matches were scheduled for the early afternoon in the United States of America (USA), corresponding to evening/night hours in other key global markets, such as Europe, Africa, and Asia. Whether the time of day influences physical performance in soccer matches is still to be determined. Moreover, environmental conditions fluctuate throughout the day, with T_a_ usually peaking in the afternoon and solar radiation being absent at night [29]. Thus, the investigation of whether the time-of-day influence on performance (if it indeed exists) is independent or dependent on environmental conditions is also warranted. The lack of monitoring environmental conditions is an important limitation of dos Santos’ study, which reported greater distances covered at night than in the afternoon [28].

Another issue overlooked in the literature is whether the clubs’ geographic origins influence their performance in hot conditions. A previous study of elite soccer found that higher game-day temperatures increase the odds of winning for national teams from warm climates when competing against national teams from cooler climates [30]. Similar findings were reported in the context of the National Football League (NFL): in matches between North (latitudes > 39°N) and South (< 39°N) teams, North teams were less likely to win and had worse point differentials as game-day temperatures increased [31]. Interestingly, the authors proposed that rather than a physiological advantage, players from teams based in warmer climates may develop behavioral and psychological adaptations (*e.g*., the application of cooling strategies and an attenuated perceptual response to hot temperatures) that can optimize performance under environmental heat stress [31]. Since these studies focused on match outcome, the effect of geographic origin on soccer clubs’ performance is yet to be investigated.

The USA hosted the 2025 FIFA Club World Cup, which featured 32 soccer teams from around the world. Matches were held under various environmental conditions, such as during heat waves; thus, data from this tournament may provide unique insights into how heat stress affects physical performance in elite male soccer. Therefore, the present study investigated whether environmental conditions influenced soccer players’ performance during official matches. We assessed the influence of factors other than T_a_ that comprise environmental conditions, as well as the effects of time of day and the geographic origin of the clubs. Our hypotheses were threefold. First, high relative humidity and solar radiation, combined with high T_a_, would impair high-speed running. Second, players would run longer distances at higher speeds in the evening than in the afternoon. Third, the performance of clubs from warm climates would be better than that of clubs from cold climates despite environmental heat stress.

## Methods

### Study design

This exploratory study investigated the relationship between environmental conditions and the players’ physical performance during the 2025 FIFA Club World Cup. Data on players’ performance were extracted from FIFA technical reports (https://www.fifatrainingcentre.com/en/game/tournaments/fcwc/2025/post-match-summary-reports.php), while data on environmental conditions were obtained through the Reanalysis v5 (ERA5) project (https://www.ecmwf.int/en/forecasts/dataset/ecmwf-reanalysis-v5).

The tournament took place in June and July, during the transition from late spring to early summer, a period that falls within the climatologically warmest part of the year. It is noteworthy that a widespread late-June heat wave affected much of the central and eastern USA, with 726 counties recording record daily maximum temperatures from June 22nd to 25th [32].

Although the tournament consisted of 63 matches, we analyzed data from 57 matches. The six matches played in Atlanta were not considered because the stadium has a retractable roof that remained closed throughout. As a result, the athletes were exposed to controlled environmental conditions, including a T_a_ of approximately 22°C (72°F).

The relationships between the environmental and performance variables were assessed using mixed-effects linear models. As all players’ technical reports were publicly available, no ethical approval was required.

### Data on the characteristics of players and teams, and their physical performance

The study included 442 professional male outfield soccer players with a mean age of 27.4 ± 4.6 years (range: 18.0 – 42.5 years). Information on players’ age was extracted from the internet, using the Google Gemini Deep Research tool (Google, Mountain View, USA). Google Gemini was prompted to run a search on each player’s name and return his date of birth in a CSV file.

All matches were analyzed using a multicamera, computer-based tracking system (Hawk-Eye Innovations Ltd, Basingstoke, United Kingdom) that is claimed to provide objective, high-precision data on match actions. It was developed by the Football Technology Center AG, a joint venture between FIFA and Hawk-Eye Innovations, and used for the first time in an international tournament. This system automatically collected most event data in real time with a frame rate of 100 Hz by tracking both players and the ball, thereby continuously monitoring player spatial positions and ball trajectories.

Analyses included individual data obtained from each player during each match. The following data were extracted from technical reports: total distance covered and distances covered at different speeds: 0 – 7 km/h (zone 1), 7 – 15 km/h (zone 2), 15 – 20 km/h (zone 3), 20 – 25 km/h (zone 4), and ≥25 km/h (zone 5). These speed zones have been used in elite soccer for more than a decade [33]. We did not analyze peak speed due to unreliable values. The sum of distances covered in zones 1 and 2 represented low-speed running, whereas the sum of distances covered in zones 4 and 5 represented high-speed running. Next, distance (in meters) was normalized by the time each athlete played (in meters per minute) for each match. For text fluency, the distance normalized by time will be named as distance throughout the manuscript.

Data from players with at least 60 min of play were used in the linear mixed-effects models; this time threshold is consistent with that adopted in previous studies [34–36]. Goalkeepers were excluded from analyses due to their distinct physical demands compared to outfield players [37]. At the end, 1070 recordings met the inclusion criteria and were utilized in our statistical analysis.

Players were grouped into three categories based on their positional roles, as described in the FIFA technical reports: defenders (DF; *n* = 175), midfielders (MF; *n* = 159), and forwards (FW; *n* = 108).

The home cities of the 32 participating teams were identified, and the Köppen-Geiger Climate Classification System was used to classify these cities’ climates as warm (*i.e*., hot desert and tropical climates; *n* = 9) or cold (other climates; *n* = 23). Annual T_a_ (mean ± SD) recorded between 2010 and 2024 corresponded to 23.9 ± 3.0°C and 14.9 ± 3.3°C in the cities located in warm and cold climates, respectively (*p* < 0.001; unpaired Student’s t-test).

### Data on environmental conditions

Data on environmental conditions were obtained through Reanalysis v5 (ERA5), the fifth-generation atmospheric reanalysis of the global climate, covering the period from January 1940 to the present. ERA5 is produced by the Copernicus Climate Change Service at the European Centre for Medium-Range Weather Forecasts (ECMWF). This reanalysis provides hourly estimates of numerous atmospheric, land, and oceanic climate variables. The data cover the Earth on a 31-kilometer grid and resolve the atmosphere into 137 levels from the surface up to a height of 80 km. More specifically, ERA5 provided data on T_a_, dew point temperature (T_d_), surface shortwave radiation flux (radiation for simplicity; R), and wind speed (W). The other meteorological variables used in the current manuscript were calculated through established equations.

The measurement of a variable at a given time was considered representative of the preceding 60 minutes. For example, if a match started at 13:00 UTC, environmental variables were collected at 14:00 and 15:00 UTC. Next, the values collected (*i.e*., at 14:00 and 15:00 UTC) were averaged to represent the environmental conditions of this specific match, thus ensuring adequate temporal resolution. In cases of extra time or prolonged interruptions due to adverse weather, data from one or two additional hours were collected. Concerning spatial resolution, we used the grid point closest to the stadium coordinates, as each point represents the spatial average within its respective grid cell. Collectively, these data with adequate temporal and spatial resolutions allowed us to estimate the heat stress athletes experienced during the matches.

The following environmental parameters were obtained at one-hour intervals: T_a_ (°C), T_d_ (°C), R (W·m^−2^), and W (m·s^−1^). W was adjusted from measurements at 10 meters to 2 meters above the surface using the following equation (Steadman [38]):

$$W=W_{10m}2100.21$$

Relative humidity (RH, %) and absolute humidity (AH, g·m^−3^) were calculated using the following sequence of equations:

$$e_{s}\left(T_{a}\right)=6.112\mathrm{ex}p^{\frac{17.67\;T_{a}}{T_{a}+243.5}}$$

$$e_{s}\left(T_{d}\right)=6.112\mathrm{ex}p^{\frac{17.67\;T_{d}}{T_{d}+243.5}}$$

$$\mathrm{RH}=100\frac{\left[e_{s}\left(T_{d}\right)\right]}{\left[e_{s}\left(T_{a}\right)\right]}$$

$$\mathrm{AH}=\frac{216.7\;e_{s}\left(T_{d}\right)}{T_{a}+273.15}$$

Natural wet-bulb temperature (T_n_, °C) and globe temperature (T_g_, °C) were estimated according to the equations applied in the study by Bitencourt et al. [39]:

$$T_{n}=0.57175\;T_{d}+0.19447\;T_{a}-0.26523\;W-0.05134\;\mathrm{RH}+10.44966$$

$$T_{g}=1.374385\;T_{a}+0.083627\;\mathrm{RH}-1.021632\;W$$

The WBGT index (°C) was calculated using the equation by Yaglou and Minard [40]:

$$\mathrm{WBGT}=0.7\;T_{n}+0.1\;T_{a}+0.2\;T_{g}$$

We next calculated the percentage of matches contested at WGBT values ≤26°C, 26–28°C, ≥28°C, ≥30°C, and ≥32°C. These thresholds were based on previous literature outlining WBGT thresholds for the risk of heat illness in soccer. The guidelines adopted by the Football Australia and the global representative organization for professional footballers (FIFPRO) indicate that: (I) a match can proceed as scheduled when WBGT is ≤26°C, (II) a cooling break in each half of a match should be implemented when WBGT is between 26°C and 28°C, and (III) a match should be delayed or postponed when WBGT is ≥28°C [41]. A 28°C WBGT threshold also corresponds to the current “permissive” upper thermal thresholds for marathon [42]. In contrast, FIFA guidelines indicate that WBGT values between 30°C and 32°C are associated with a high risk of heat illness, while values ≥32°C are associated with extreme risk [41]. Indeed, a medical report issued by FIFA states that if the WBGT reading is ≥32°C, cooling breaks are mandatory, or the match may be postponed or canceled [43]. Because FIFA guidelines appear less protective than those in other sports or in countries traditionally exposed to extreme hot conditions, we adopted more conservative guidelines and classified WBGT ≥28°C as extreme heat.

### Statistical analysis

Normality of residuals and random effects for all linear models was assessed graphically and using the Kolmogorov-Smirnov test, while homoscedasticity was assessed graphically (data presented in the supplementary material: https://github.com/Lafise-UFMG/Supplementary-material-linear-mixed-effects-model-fcwf-2025.git). All results are reported as means ± standard deviations (SDs), unless otherwise specified. Unpaired Student’s t-tests were used to compare environmental conditions (T_a_, T_d_, T_g_, T_n_, WBGT, RH, AH, R, and W) between matches played in the afternoon and in the evening.

Four different linear mixed-effects models were fitted to assess the effects of environmental conditions on sprint (zone 5), high-speed (zones 4 and 5), moderate-speed (zone 3), and low-speed (zones 1 and 2) running per minute, as well as on total distance covered per minute during the matches. Data regarding sprints (zone 5) markedly deviated from a normal distribution and violated homoscedasticity assumption and, therefore, were not presented in the current manuscript (Figures S21 to S24 and tables S49 and S50 in the supplementary material).

Players were modeled as a random effect. Fixed effects included contextual and individual factors already known to influence physical performance, such as the opponent quality [44], player position [45], and player age [46]. Opponent quality was addressed through two different factors: the tournament stage (group stage vs. playoffs) and the difference in final classification. These fixed effects were included as positive controls in our analysis. Other fixed effects of interest were also included, such as the time of day (afternoon or evening) and the geographic origin of the club (warm vs. cold climates), as justified in the Introduction section. Moreover, different environmental variables were entered into each model, as detailed in Table 1.

**Table 1.** Four different linear mixed-effects models assessed the effects of environmental conditions on players’ physical performance.

Model	Fixed effects
All	tournament stage + time of day + geographic origin + difference in classification + player’s position + player’s age +
1	+ wet-bulb globe temperature (WBGT)
2	+ air temperature (T_a_) + relative humidity (RH) + solar radiation (R)
3	+ air temperature (T_a_) + absolute humidity (AH) + solar radiation (R)
4	+ air temperature (T_a_) + wet-bulb temperature (T_n_) + globe temperature (T_g_)

Notably, model 3 considers AH because this meteorological variable (not RH) governs evaporative rate [47]. Model 4 includes the three temperatures required to calculate WGBT (model 1), but removes the weights assigned to these temperatures during that calculation.

Numeric variables – player’s age, T_a_, T_g_, T_n_, AH, RH, WBGT, and R – were mean-centered by subtracting the variable’s mean from each observation. This procedure made the model’s intercepts more meaningful and improved interpretability. Collinearity among independent variables was analyzed using the variance inflation factor (*i.e*., VIF > 10 indicated collinearity). Tukey’s post hoc tests were conducted to determine if there were significant differences between player positions. Conditional R^2^ was calculated to describe the variance explained by both fixed and random effects, and marginal R^2^ was calculated to describe the variance explained only by fixed effects. Models were compared using the Akaike information criterion (AIC); the model with the lowest AIC value was considered the most parsimonious. The statistical significance of individual fixed effects was assessed using p-values.

Statistical analyses were performed using RStudio software version 2025.05.0 Build 496© 2009–2025 Posit Software, PBC, using R version 4.5.0. Mixed-effects linear analysis was performed using the lme4 and lmerTest packages. The level of statistical significance was set at *p* < 0.05.

## Results

### Dataset characterization

The dataset analyzed included 1070 observations, of which 436, 391, and 243 were obtained from defenders, midfielders, and forward players, respectively. Moreover, the dataset included different numbers of observations for the tournament stage (group stage matches: 833 vs. playoffs matches: 237), geographic origin (warm climates: 316 vs. cold climates: 754), and time of day (afternoon: 618 vs. evening: 452).

The mean total distance covered by the players was 104.0 ± 10.8 m/min. Of this distance, players covered, on average, 7.3 ± 2.7 m/min at high speeds, 13.7 ± 4.15 m/min at moderate speeds, and 83.0 ± 6.92 m/min at low speeds.

The data regarding environmental conditions during the 57 matches are summarized in Table 2. All variables had high CVs (≥15.2%), indicating that matches occurred across diverse environmental conditions. Notably, solar radiation was the most heterogeneous variable (CV = 85.1%). SL Benfica × FC Bayern München (played at 3 pm, Charlotte) occurred at the hottest T_a_ (37.4°C) and higher WBGT (32.9°C), whereas FC Internazionale Milano × Urawa Red Diamonds (played at 12 pm, Seattle) occurred at the coldest T_a_ (12.7°C) and lower WBGT (15.8°C).

**Table 2.** Environmental conditions during the 57 matches of the 2025 FIFA Club World Cup.

Variable	Mean	SD	CV (%)	Min	Max
Air temperature (T_a_, °C)	27.0	5.1	18.8	12.7	37.4
Dew point temperature (T_d_,°C)	18.7	5.0	26.9	7.3	25.6
Wet-bulb temperature (T_n_,°C)	22.8	3.5	15.2	14.1	26.9
Globe temperature (T_g_,°C)	40.7	6.6	16.2	23.0	52.6
WBGT (°C)	26.8	4.2	15.6	15.8	32.9
Relative humidity (RH,%)	61.9	15.6	25.3	24.5	93.0
Absolute humidity (AH, g·m^−3^)	26.6	7.0	26.2	11.2	44.8
Solar radiation (R, W·m^−2^)	350	298	85.1	0	1035
Wind speed (W, m·s^−1^)	1.6	0.7	41.0	0.3	3.1

Legend: CV = coefficient of variation; Max = maximum value recorded; Min = minimum value recorded; SD = standard deviation; WBGT = wet-bulb globe temperature.

Mean WBGT values were ≤26°C in 17 (29.8%) of the 57 matches analyzed. In addition, mean WBGT was above 26°C and below 28°C in 9 (15.8%) matches, but it was ≥28°C in 31 (54.4%) matches. Importantly, 13 and 2 matches were contested with mean WBGT ≥30°C and 32°C, respectively. Considering only the first hour, when the values of environmental variables were increased compared to the second and subsequent hours, WBGT was ≥30°C and 32°C in 16 and 4 matches, respectively.

Thirty-three (57.9%) matches occurred in the afternoon (*i.e*., they started at 5 pm or earlier). The remaining 24 matches (42.1%) occurred in the evening (starting at 6 pm or later). T_a_, T_g_, WBGT, AH, and R were lower in the evening than in the afternoon (Table 3). RH was the only variable that was higher in the evening. No significant differences in T_d_, T_n_, and W were observed across times of day.

**Table 3.** Comparison of environmental conditions between matches occurring in the afternoon and in the evening.

Variable	Afternoon (*n* = 33)	Evening (*n* = 24)	*p*-value
Air temperature (T_a_, °C)	28.5 ± 5.2	25.0 ± 4.2	0.008
Dew point temperature (T_d_,°C)	19.0 ± 4.9	18.3 ± 5.3	0.607
Wet-bulb temperature (T_n_,°C)	23.4 ± 3.5	21.8 ± 3.3	0.085
Globe temperature (T_g_,°C)	42.5 ± 6.7	38.3 ± 5.7	0.018
WBGT (°C)	27.8 ± 4.2	25.4 ± 3.8	0.039
Relative humidity (RH,%)	57.9 ± 15.2	67.5 ± 14.7	0.021
Absolute humidity (AH, g·m^−3^)	28.9 ± 7.2	23.5 ± 5.3	0.003
Solar radiation (R, W·m^−2^)	553 ± 223	70 ± 77	<0.001
Wind speed (W, m·s^−1^)	1.6 ± 0.7	1.7 ± 0.7	0.722

Data are expressed as means ± SDs. Legend: WBGT = wet-bulb globe temperature.

### Statistical modeling of physical performance

**Figure 1.**
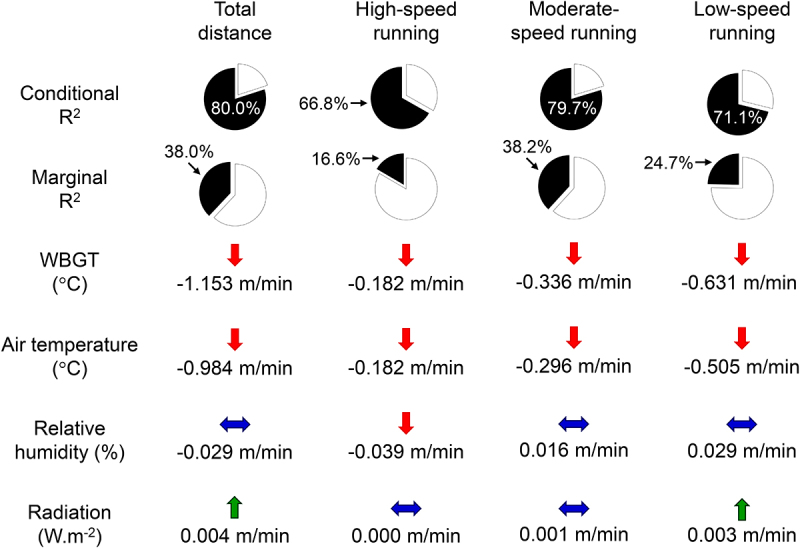
Conditional and marginal R^2^ values for the best statistical model fitted from 1070 observations for each performance variable: total distance covered (model 2) and distances covered at high (model 1), moderate (model 1), and low speeds (model 1). The figure also shows the effects of a 1°C increase in WBGT and air temperature, a one percentage point increase in relative humidity, and a 1 W.m^−2^ increase in radiation on the distances players covered. Red and green arrows indicate significant reductions and increases, respectively; blue arrows indicate non-significant effects. The changes in distance correspond to values when the time of day was set to the reference level (afternoon). Please note that it is not possible to compare the magnitudes of changes in distance across the different environmental variables due to differences in measurement units.

Of the four linear mixed-effects models tested, the findings of models 3 and 4 were artificially inflated by unacceptable collinearity between independent variables: T_a_ and AH in model 3; T_a_, T_n_, and T_g_ in model 4 (VIFs substantially above 10, as demonstrated in supplementary tables S41 to S48). Since these two models also exhibited collinearity in analyses of distances covered at different speeds, they were not further considered in the current manuscript. The remaining two models tested for each performance variable had similar AIC values, hampering the identification of the best model (Tables 4–7). Conditional R^2^ values ranged from 0.668 (distance at high speeds, model 1) to 0.801 (total distance, model 1), whereas marginal R^2^ values ranged from 0.166 (distance at high speeds, model 1) to 0.383 (total distance, model 1). These discrepant conditional and marginal R^2^ values indicate that a significant percentage of the variance is explained by factors not assessed in our model, such as individual differences (Figure 1).

**Table 4.** Fixed effects estimates and model statistics of the two mixed-effects linear models elaborated to explain total distance covered during the 2025 FIFA Club World Cup matches.

Variables	Model 1		Model 2	
	Estimate [95%CI]	*p*-value	Estimate [95%CI]	*p*-value
Intercept	96.937 [95.177, 98.696]	<0.001	96.327 [94.482, 98.173]	<0.001
Stage (playoffs)	−0.805 [−1.777, 0.168]	0.105	−1.038 [−2.117, 0.040]	0.059
Time of day (evening)	1.963 [1.150, 2.776]	<0.001	3.424 [1.870, 4.977]	<0.001
Difference in classification	0.000 [−0.035, 0.034]	0.980	0.010 [−0.025, 0.044]	0.581
Player position (midfielder)	9.667 [7.976, 11.358]	<0.001	9.693 [8.010, 11.376]	<0.001
Player position (forward)	4.586 [2.685, 6.488]	<0.001	4.600 [2.707, 6.493]	<0.001
Player age (centered)	−0.279 [−0.443, −0.116]	<0.001	−0.285 [−0.447, −0.122]	<0.001
Origin (cold climates)	3.008 [1.349, 4.668]	<0.001	3.080 [1.425, 4.734]	<0.001
WBGT (centered)	−1.153 [−1.284, −1.022]	<0.001	NA	NA
T_a_ (centered)	NA	NA	−0.984 [−1.118, −0.850]	<0.001
RH (centered)	NA	NA	−0.029 [−0.077, 0.019]	0.235
R (centered)	NA	NA	0.004 [0.001, 0.007]	0.010
*Interactions*				
Time of day × WBGT	0.421 [0.236, 0.606]	<0.001	NA	NA
Time of day × T_a_	NA	NA	0.386 [0.214, 0.559]	<0.001
Time of day × RH	NA	NA	0.011 [−0.046, 0.068]	0.714
*Model statistics*				
Conditional R^2^	0.801		0.800	
Marginal R^2^	0.383		0.380	
AIC	7202		7199	

Legend: AIC = Akaike information criterion; NA = not addressed; *R* = radiation; RH = relative humidity; T_a_ = air temperature; WBGT = wet-bulb glob temperature.

**Table 5.** Fixed effects estimates and model statistics of the two mixed-effects linear models elaborated to explain high-speed running during the 2025 FIFA Club World Cup matches.

Variables	Model 1		Model 2	
	Estimate [95% CI]	*p*-value	Estimate [95% CI]	*p*-value
Intercept	6.562 [6.071, 7.053]	<0.001	6.580 [6.057, 7.102]	<0.001
Stage (playoffs)	0.021 [−0.276, 0.319]	0.889	0.090 [−0.239, 0.419]	0.591
Time of day (evening)	0.382 [0.132, 0.632]	0.003	0.286 [−0.194, 0.766]	0.244
Difference in classification	0.004 [−0.006, 0.014]	0.642	0.005 [−0.006, 0.015]	0.364
Player position (midfielder)	0.600 [0.134, 1.066]	0.012	0.597 [0.131, 1.063]	0.012
Player position (forward)	1.506 [0.981, 2.030]	<0.001	1.508 [0.983, 2.032]	<0.001
Player age (centered)	−0.087 [−0.132, −0.042]	<0.001	−0.086 [−0.131, −0.041]	<0.001
Origin (cold climates)	0.041 [−0.417, 0.499]	0.860	−0.008 [−0.467, 0.451]	0.973
WBGT (centered)	−0.182 [−0.222, −0.142]	<0.001	NA	NA
T_a_ (centered)	NA	NA	−0.182 [−0.222, −0.141]	<0.001
RH (centered)	NA	NA	−0.039 [−0.054, −0.025]	<0.001
R (centered)	NA	NA	0.000 [−0.001, 0.001]	0.499
*Interactions*				
Time of day × WBGT	0.007 [−0.050, 0.064]	0.812	NA	NA
Time of day × T_a_	NA	NA	0.019 [−0.035, 0.072]	0.495
Time of day × RH	NA	NA	0.026 [0.008, 0.044]	0.004
*Model statistics*				
Conditional R^2^	0.668		0.673	
Marginal R^2^	0.166		0.175	
AIC	4609		4610	

Legend: AIC = Akaike information criterion; NA = not addressed; *R* = radiation; RH = relative humidity; T_a_ = air temperature; WBGT = wet-bulb glob temperature.

**Table 6.** Fixed effects estimates and model statistics of the two mixed-effects linear models elaborated to explain moderate-intensity distance during the 2025 FIFA Club World Cup matches.

Variables	Model 1		Model 2	
	Estimate [95% CI]	*p*-value	Estimate [95% CI]	*p*-value
Intercept	10.941 [10.266, 11.617]	<0.001	10.750 [10.036, 11.464]	<0.001
Stage (playoffs)	−0.525 [−0.902, −0.147]	0.007	−0.521 [−0.941, −0.101]	0.015
Time of day (evening)	0.529 [0.254, 0.885]	0.161	1.026 [0.420, 1.631]	<0.001
Difference in classification	0.007 [−0.007, 0.020]	0.318	0.009 [−0.004, 0.023]	0.183
Player position (midfielder)	4.484 [3.835, 5.132]	<0.001	4.487 [3.837, 5.137]	<0.001
Player position (forward)	2.150 [1.420, 2.879]	<0.001	2.153 [1.422, 2.885]	<0.001
Player age (centered)	−0.063 [−0.125, 0.000]	0.050	−0.064 [−0.127, −0.001]	0.046
Origin (cold climates)	0.753 [0.116, 1.389]	0.021	0.754 [0.115, 1.393]	0.021
WBGT (centered)	−0.336 [−0.387, −0.285]	<0.001	NA	NA
T_a_ (centered)	NA	NA	−0.296 [−0.348, −0.243]	<0.001
RH (centered)	NA	NA	0.016 [−0.035, 0.002]	0.087
R (centered)	NA	NA	0.001 [0.000, 0.002]	0.057
*Interactions*				
Time of day × WBGT	0.026 [−0.046, 0.097]	0.486	NA	NA
Time of day × T_a_	NA	NA	0.026 [−0.041, 0.094]	0.442
Time of day × RH	NA	NA	−0.003 [−0.025, 0.019]	0.802
*Model statistics*				
Conditional R^2^	0.797		0.797	
Marginal R^2^	0.382		0.380	
AIC	5168		5176	

Legend: AIC = Akaike information criterion; NA = not addressed; *R* = radiation; RH = relative humidity; T_a_ = air temperature; WBGT = wet-bulb glob temperature.

**Table 7.** Fixed effects estimates and model statistics of the two mixed-effects linear models elaborated to explain low-intensity distance during the 2025 FIFA Club World Cup matches.

Variables	Model 1		Model 2	
	Estimate [95%CI]	*p*-value	Estimate [95%CI]	*p*-value
Intercept	79.460 [78.218, 80.701]	<0.001	78.997 [77.700, 80.295]	<0.001
Stage (playoffs)	−0.378 [−1.115, 0.360]	0.316	−0.660 [−1.466, 0.147]	0.109
Time of day (evening)	0.956 [0.337, 1.575]	0.003	2.115 [0.940, 3.290]	<0.001
Difference in classification	−0.009 [−0.035, 0.017]	0.485	−0.003 [−0.029, 0.023]	0.819
Player position (midfielder)	4.603 [3.421, 5.785]	<0.001	4.629 [3.467, 5.790]	<0.001
Player position (forward)	0.927 [−0.404, 2.259]	0.173	0.934 [−0.373, 2.241]	0.162
Player age (centered)	−0.133 [−0.247, −0.018]	0.023	−0.138 [−0.251, −0.026]	0.016
Origin (cold climates)	2.232 [1.070, 3.394]	<0.001	2.358 [1.214, 3.501]	<0.001
WBGT (center)	−0.631 [−0.731, −0.532]	<0.001	NA	NA
T_a_ (centered)	NA	NA	−0.505 [−0.605, −0.406]	<0.001
RH (centered)	NA	NA	0.029 [−0.007, 0.066]	0.112
R (centered)	NA	NA	0.003 [0.001, 0.005]	0.004
*Interactions*				
Time of day × WBGT	0.398 [0.256, 0.539]	<0.001	NA	NA
Time of day × T_a_	NA	NA	0.349 [0.219, 0.480]	<0.001
Time of day × RH	NA	NA	−0.014 [−0.057, 0.029]	0.527
*Model statistics*				
Conditional R^2^	0.712		0.711	
Marginal R^2^	0.241		0.247	
AIC	6563		6538	

Legend: AIC = Akaike information criterion; NA = not addressed; *R* = radiation; RH = relative humidity; T_a_ = air temperature; WBGT = wet-bulb glob temperature.

*Player age* had a statistically significant detrimental effect on the total distance covered and distances at different running speeds (*p* ≤ 0.05; Tables 4–7). For example, results from model 1 showed that for each year increase in age, the total distance covered decreased by 0.279 m/min (95% CI: −0.443, −0.116), and the distance covered at high speeds decreased by 0.087 m/min (95% CI: −0.132, −0.042).

*Player field position* also significantly contributed to explaining the variance in the total distance and distances at different running speeds (Tables 4–7). Midfielders and forwards covered greater total distances (*p* < 0.001) and also greater distances in high and moderate speeds than defenders (*p* ≤ 0.012). Considering low speeds, midfielders covered greater distances than defenders (*p* < 0.001), although no significant differences were observed between forwards and defenders (*p* ≥ 0.162).

The *difference in final classification* between teams did not significantly predict the variance in total distance covered (*p* ≥ 0.581; Table 4) nor in the distances covered at high (*p* ≥ 0.364; Table 5), moderate (*p* ≥ 0.183; Table 6), or low speeds (*p* ≥ 0.485; Table 7). The *tournament stage* did not significantly explain the variance in the total distance or distances at high and low speeds (*p* ≥ 0.059; Tables 4, 5 , and 7). However, shorter distances were covered at moderate speeds during the playoffs compared to the group stage (Table 6). For example, results from model 1 showed that players covered 0.525 m/min (95% CI: −0.902, −0.147) less at moderate speeds during the playoffs than in the group stage (*p* = 0.007).

The *geographic origin* of the clubs significantly predicted the variance in total distance covered (*p* < 0.001; Table 4), as well as the distances covered at moderate (*p* = 0.021; Table 6) and low speeds (*p* < 0.001; Table 7). For instance, results from model 2 showed that players from clubs based in cold climates ran longer distances (3.080 m/min; 95% CI: 1.425, 4.734) than those from clubs based in warm climates. However, geographic origin did not significantly predict the distance at high speeds (*p* ≥ 0.860; Table 5).

**Figure 2.**
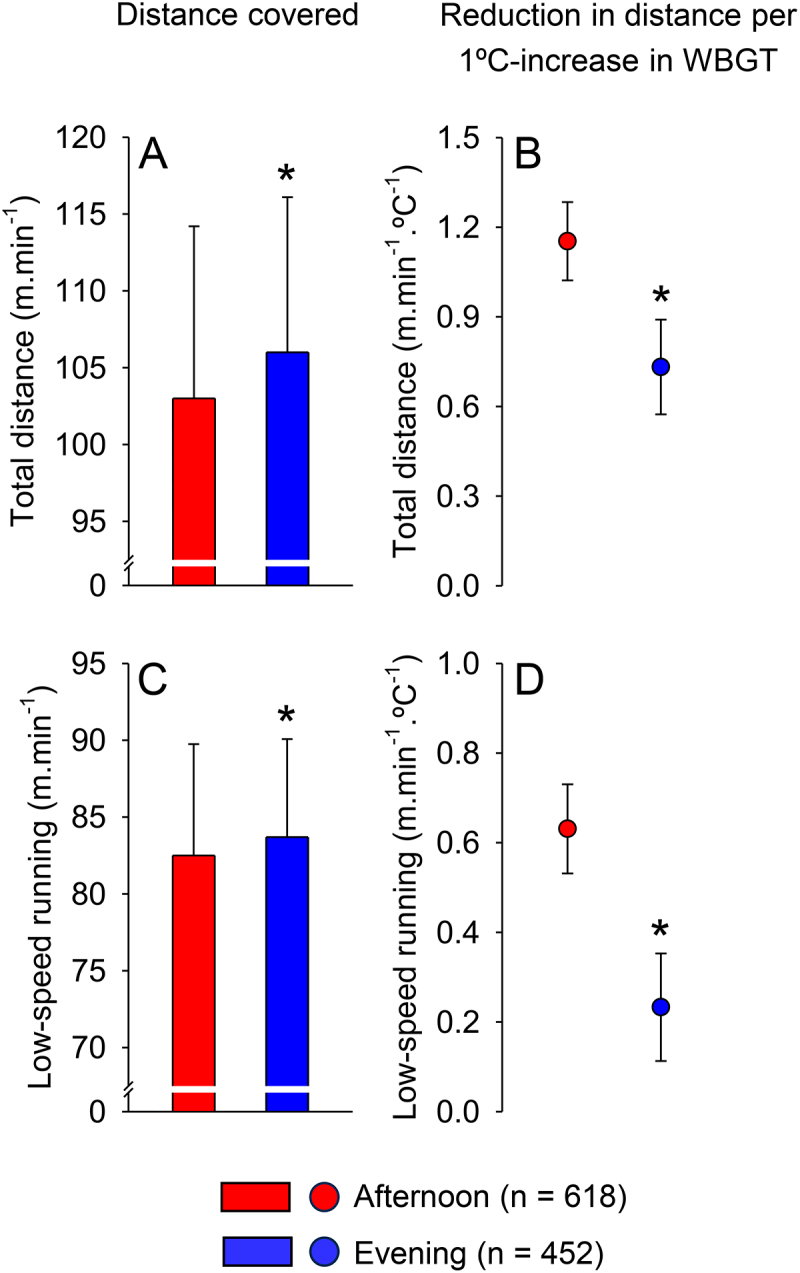
Total (panel A) and low-speed distances (panel C) during matches in the afternoon (*n* = 618 observations) and in the evening (*n* = 452 observations). The distance values correspond to those when the mean wet-bulb globe temperature (WBGT) was 26.8°C (*i.e*., WBGT average value for the analyzed data set). Data are expressed as means ± SDs. Significant interactions between time of day and WBGT were observed for total (panel B) and low-speed distances (panel D). These panels show the reduction in distance covered per 1°C increase in WBGT, both in the afternoon and in the evening. Data are expressed as means ± 95% confidence intervals. *indicates a significant difference (*p* < 0.05) from the afternoon.

*Time of day* significantly affected total (*p* < 0.001; Table 4) and low-speed distances (*p* ≤ 0.003; Table 7). In addition, time of day demonstrated a significant main effect in at least one model for distance covered at high (model 1, *p* = 0.003; Table 5) and moderate speeds (model 2, *p* < 0.001; Table 6). Because significant interactions between time of day and several environmental variables were observed (see below), the numbers presented in the following comparison and in Figure 2 are valid only when considering the mean values of these environmental variables in our dataset. For example, the total distance covered was 1.963 m/min higher (95% CI: 1.150, 2.776) in the evening than in the afternoon, when the mean WBGT corresponded to 26.8°C.

*WBGT* showed a statistically significant detrimental effect on the distances covered across all running speeds when time of day was at the reference level (afternoon) (*p* < 0.001; Tables 4–7 and Figure 1). For example, for each 1°C increase in WBGT, players decreased distance at high speeds by 0.182 m/min (95% CI: −0.222, −0.142; Table 5) and at moderate speeds by 0.336 m/min (95% CI: −0.387, −0.285; Table 6). Significant interactions between WBGT and time of day were observed for total (*p* < 0.001; Table 4) and low-speed distances (*p* < 0.001; Table 7). Thus, for each 1°C increase in WBGT, players decreased the total distance covered by 1.153 m/min (95% CI: −1.284, −1.022) in the afternoon and by 0.732 m/min (95% CI: −0.890, −0.573) in the evening.

*Air temperature* (T_a_) significantly predicted distances covered across all intensities when time of day was fixed at the reference level (*p* < 0.001; Tables 4–7 and Figure 1). Players decreased distance at high speeds by 0.182 m/min (95% CI: −0.222, −0.141; Table 5) and at moderate speeds by 0.296 m/min (95% CI: −0.348, −0.243; Table 6) for each 1°C increase in T_a_. Significant interactions were also observed between T_a_ and time of day for total (*p* < 0.001; Table 4) and low-speed distances (*p* < 0.001; Table 7). For example, for each 1°C increase in T_a_, players decreased the total distance covered by 0.984 m/min (95% CI: −1.118, −0.85) in the afternoon and by 0.598 m/min (95% CI: −0.740, −0.457) in the evening.

A statistically significant detrimental effect of *relative humidity* (RH) was observed for distance covered at high speeds (*p* < 0.001; Table 5) but not for total distance (*p* = 0.235; Table 4) or distances at moderate (*p* = 0.087; Table 6) and low speeds (*p* = 0.112; Table 7). A significant interaction between time of day and RH was observed only for distance at high speeds (*p* = 0.004; Table 5). For each 1% point (p.p.) increase in RH, players covered 0.039 m/min (95% CI: −0.054, −0.025) less in the afternoon and 0.013 m/min (95% CI: −0.027, −0.000) less in the evening.

Radiation (R) significantly affected the total distance (*p* = 0.010; Table 4) and distance at low speeds (*p* = 0.004; Table 7). However, R was not a significant predictor of distance at high speeds (*p* = 0.499; Table 5) and was marginally significant at moderate speeds (*p* = 0.057; Table 6). For instance, for each 1 W.m^−2^ increase in R, total distance covered increased by 0.004 m/min (95% CI: 0.001, 0.007), while distance at low speeds increased by 0.003 m/min (95% CI: 0.001, 0.005).

## Discussion

The current study identified several factors that explained the total distance and the distance covered at different speeds during the 2025 FIFA Club World Cup. Our analysis emphasized the role played by environmental factors. The main findings were that total distance and distances at different speeds were shorter with increasing WBGT or T_a_, whereas only distance at high speeds was shorter with increasing relative humidity. These findings confirm our first hypothesis and highlight the importance of environmental conditions in determining athletes’ physical performance during an international tournament held in the summer in the Northern Hemisphere. Of note, factors other than environmental conditions also influenced the distances covered by the players, including their position and age, time of day, and the club’s geographic origin.

WBGT and T_a_ consistently affected physical performance: the higher the WBGT (or T_a_), the lower the distances covered by players at all analyzed speeds, including at high speeds, during the 2025 FIFA Club World Cup. This finding corroborates previous investigations showing that high-speed running was markedly reduced in the heat [18–22,24]. Since the decisive actions of a soccer match commonly occur at high intensities [23], environmental heat stress can significantly influence the match outcome. The mechanism underlying reduced performance in the heat may involve accentuated body hyperthermia, as evidenced by elevated core and muscle temperatures during matches in hotter environments [18]. Marked hyperthermia alters individual behavior during prolonged self-paced running [48,49] and cycling [50] in the heat, leading athletes to select lower exercise intensities. This explanation may also apply to soccer matches, as evidenced by players running less at higher speeds, especially during the first half, and choosing a possession-oriented over a transition-based style of play at 43°C compared to 21°C [18]. A behavioral change in the game style due to augmented heat strain is also supported by a recent study that reported significantly fewer total passes and passes into the offensive final third of the pitch at higher WBGT or T_a_, depicting a more static, slower match play [51].

High relative humidity decreased the distance covered at high speeds, tended to increase distance at moderate speeds (*p* = 0.087), but did not change the low-speed or total distance. The fact that high relative humidity reduced the distance covered at high speeds, which is decisive for match outcome [23], highlights the importance of this factor in regulating soccer’s physical performance. Moreover, the trend toward greater distance at moderate speeds with increasing humidity suggests that players adjusted their pace and/or made tactical decisions (*e.g*., adopted a more ball-possession-oriented strategy) in response to the challenging environmental conditions.

In previous investigations into environmental conditions and soccer, relative humidity was indirectly assessed by calculating WBGT. Therefore, the current findings are novel and can be compared only with those in other sports. For example, hot and humid conditions greatly impair tolerance to prolonged fixed-intensity cycling to exhaustion [25] and reduce power output during self-paced cycling [52] compared with hot and dry conditions. Under hot conditions, the temperature gradient between the body and the environment becomes narrow, making evaporation the primary mechanism for heat dissipation [53]. When combined with humid conditions, the detrimental effects of high T_a_s on body heat exchange are more pronounced. Higher humidity reduces the gradient in partial water vapor pressure between the skin and the surrounding air, which significantly impairs the body’s ability to dissipate heat through evaporation [53]. In this sense, Bright et al. [52] reported that reductions in the maximum evaporative capacity of the environment and in sweating evaporative efficiency resulting from elevated humidity exacerbated the increases in core and skin temperatures induced by self-paced cycling in the heat, thereby amplifying thermal and perceptual strain and impairing endurance performance.

Greater radiation levels were associated with longer distances run at low speeds, resulting in a higher total distance covered during the match. However, because increased distance was observed at low, but not at moderate or high speeds, we interpret that radiation had no significant effect on physical performance under the conditions currently studied. This finding contradicts previous research indicating that solar radiation and high T_a_ combine to decrease aerobic performance [26,54], due to increased physiological and perceptual strain, as evidenced by higher skin temperature values and thermal sensation scores, narrower core-to-skin temperature gradient, and augmented body heat gain with increasing solar radiation. Indeed, care should be taken when interpreting our findings, as many World Cup stadiums are multi-level structures that limit solar radiation on parts of the soccer pitch in the afternoon and early evening, creating shaded areas. Future studies conducted in training centers, which usually lack large structures around the soccer fields, may be relevant for identifying the effects of solar radiation on athletes’ physical performance.

Time of day explained the distances covered by the athletes in most models tested (6 of 8), with athletes running longer distances in the evening than in the afternoon, which confirms our second hypothesis. A simple explanation is that evening matches were contested under lower environmental heat stress than afternoon matches. Indeed, WBGT, T_a_, globe temperature, and radiation were all lower in the evening. Nevertheless, our analysis did not identify consistent interactions between time of day and environmental variables. Significant interactions were observed with WBGT (or T_a_) for the total and low-speed distances, and with relative humidity for high-speed distance. The lack of consistent interactions suggests that unidentified factors, including chronobiological ones, could influence soccer players’ performance at different times of the day. While the current findings agree with those reported for a fourth-division Brazilian team [28], they contradict a laboratory study showing that the time to fatigue during a fixed-speed run was more prolonged in the morning than at night [55]. Moreover, since spectators reported greater perceptual strain when watching a simulated match in hot, humid conditions [56], it cannot be ruled out that their support is more effective in encouraging players during evening matches.

The clubs’ geographic origins did not explain high-speed running, but they did influence the distances at other speeds. Indeed, players from clubs based in cold climates ran more at lower and moderate speeds, covering a greater total distance than those from clubs based in warm climates. These observations refute our third hypothesis that clubs from warm climates would cover longer distances. The following aspects help explain the unexpected findings. First, athletes from clubs based in warm climates might have experience in pacing their exertion in the heat [30] and therefore covered shorter distances at moderate and low intensities to preserve their ability to exercise at high intensity. Second, a broad knowledge of heat mitigation strategies is available [57], so that clubs from cold climates can also employ these effective strategies when playing under environmental heat stress. Moreover, cities not located in tropical or hot desert climates are also affected by environmental conditions that produce significant heat stress, which can favor the development of beneficial behavioral and psychological adaptations to tolerate these adverse conditions. For example, southern regions of the Brazilian territory have experienced more frequent and intense heat waves than the northern regions in recent decades [5].

Player position consistently influenced the distance players ran. The defenders always ran less than the other player positions, except for the distance covered at low speeds, which was comparable to that of forwards. In addition, midfielders ran greater total distances and distances at moderate and low speeds than defenders and forwards, while forwards ran longer distances than midfielders at high speeds. These findings partially agree with previous literature. A recent systematic review, which classified players into five field positions, found that central midfielders, external midfielders, and external defenders cover greater total and high-speed distances than forwards or central defenders [45]. Thus, the main discrepancy between the data reported in the present study and the 2024 study concerns the distance covered by forwards at high speeds: the FIFA Club World Cup data indicate that forwards run longer distances at high speeds than midfielders, contradicting Sarmento et al. [45]. These differences between studies may be explained by FIFA's division of players into only three field positions, treating central and external midfielders as a single position. Another reason is that teams have been more often employing a high-pressure strategy to recover the ball on the offensive field, thus requiring the forwards to run at higher intensities and perform more accelerations and decelerations [58].

Our analysis indicated players’ age as a significant explanatory factor. The older the player was, the less he ran in all speeds investigated, thus leading to a lower total distance covered. This finding aligns with previous observations of players competing in the Spanish first division (*i.e*., La Liga), where soccer players, on average, decreased their total distance covered by 0.56% per year as they aged [46]. Moreover, the distance at high speeds decreased by 1.42% per year [46]. Thus, robust evidence indicates that age is a relevant factor in determining physical performance in soccer matches. However, the interaction of age with environmental heat stress should be explored in future studies.

We expected that stronger opponent teams would force players to run farther distances, as recently reported for the English Premier League and Championship League matches [44]. For this purpose, we included two contextual factors in our analyses: tournament stage (group stage vs. playoffs) and the difference in classification between teams. The tournament stage explained the distance covered only at moderate speeds, with players running less in playoff matches than in group-stage matches. In addition, the ranking differences did not significantly explain the dependent variable in any of the models tested. Altogether, these findings from the 2025 FIFA Club World Cup contradict the notion that better-ranked opponents force players to run more.

The present findings are unique because the database used contains matches played under very adverse conditions, unlike previous investigations that comprised matches at colder temperatures [24]. For example, 31 and 2 matches occurred at mean WBGT values ≥28°C and ≥32°C, respectively. According to the heat guidelines of Football Australia and FIFPRO, a match should be delayed or postponed when WBGT is ≥28°C [41], indicating that almost half of the 61 tournament matches were held under extreme heat. Although FIFA guidelines are more permissive than those of Football Australia and FIFPRO [41], four matches were started with a mean WBGT ≥32°C, despite FIFA’s mention of the possibility of postponing or canceling a match [43]. Given that the global warming trend is unlikely to be reversed even with reductions in greenhouse gas emissions [59], the current findings underscore the urgency of developing more efficient strategies to ensure players’ performance and health. In addition, the current findings are highly relevant for the preparation of national teams for the 2026 FIFA World Cup, which be held in North America (Canada, Mexico, and the USA), since predictions indicate that ten of sixteen venues will very likely experience severe heat stress conditions [60].

This study is not without limitations. First, environmental condition data were obtained through mathematical modeling rather than onsite measurement. Previous evidence showed station-derived WBGT values underestimated or overestimated the onsite WBGT values by >3°C in 45% of paired comparisons during the Boston Marathon race [61]. Similarly, WBGT values obtained from weather stations underestimated those measured on nearby fields of play on a university campus in 84% to 92% of cases, resulting in clinically meaningful differences [62]. Further, the local incidence of solar radiation depends on the cloud cover. Therefore, variations in the local cloud cover may not have been adequately accounted for in our simulations during the matches. This limitation is minimized because matches were played in the afternoon and at night, which improves reanalysis performance, as night matches occurred mainly when solar radiation was absent or low.

Second, although our statistical models could explain the dependent variables, the weak and moderate correlations between the independent and dependent variables suggest that the data may be better modeled using non-linear statistical models. Linear models were initially chosen for simplicity of analysis and interpretability. However, considering that the detrimental effects of environmental conditions on physical performance may increase more steeply at higher values of the environmental variables tested, future work should explore non-linear modeling techniques. Third, the data analyzed were obtained from the FIFA website; therefore, some important indicators of physical performance, such as the number of accelerations and decelerations, were unavailable. Fourth, FIFA classified players into only three field positional roles (*i.e*., defenders, midfielders, and forwards). As reviewed recently by Sarmento et al. [45], most studies classify players into five positions: central defenders, external defenders, central midfielders, external midfielders, and forwards. Fifth, cooling breaks were implemented in some matches of the 2025 FIFA Club World Cup. However, the data we accessed from the FIFA website does not differentiate the distances covered by each player before and after the 3-minute match interruptions; therefore, we could not assess the impact of cooling breaks on athletes’ performance in this tournament.

In conclusion, environmental heat stress significantly affects the physical performance of soccer players, as further highlighted by data from the 2025 FIFA Club World Cup. Among the environmental factors analyzed, air temperature and relative humidity play prominent roles in determining performance, especially the distance covered at high speeds. WBGT, an index that measures environmental heat stress, also explains performance during soccer matches.

The current findings confirm the roles of players’ age and field position, and provide preliminary evidence for the roles of clubs’ geographic origins and time of day in regulating physical performance. Notably, the influence of time of day is not fully explained by differences in environmental conditions. Collectively, these findings highlight the multifaceted regulation of physical performance in soccer players and emphasize the role of environmental conditions in determining distances covered at different speeds.

From an applied perspective, the current findings emphasize the importance of adopting heat mitigation strategies to protect elite soccer players’ performance and health. These strategies can be broad and include advanced planning of match hours and choosing host cities that are not commonly affected by extreme weather events during the period/season of a given tournament. For example, matches should not be scheduled for the early afternoon when WBGT and T_a_ are high, and a retrospective analysis of historical environmental data should help identify cities that are more often affected by extreme events. Soccer-specific heat acclimation protocols should be developed and, if deemed effective, encouraged. Along the same lines, although evidence indicates that FIFA cooling breaks attenuated physiological and perceptual strain in the heat either during real soccer matches [63] or simulated matches in the laboratory [64], there is still room to improve the effectiveness of cooling and hydration procedures, particularly at half-time [63]. Finally, coaches should be aware of how environmental conditions affect performance and adapt their game plan to the match hour and expected conditions. When environmental heat stress is anticipated, coaches may select a possession-oriented style of play, employ a high-pressure strategy (to recover the ball) less frequently, and make more early substitutions during the match.

## Abbreviations

AH: absolute humidity
AIC: Akaike information criterion
CV: coefficient of variation
FIFA: Fédération Internationale de Football Association
FIFPRO: Fédération Internationale des Associations de Footballeurs Professionnels
Max: maximum value recorded
Min: minimum value recorded
NA: not addressed
R: solar radiation
RH: relative humidity
SD: standard deviation
T_a_: air temperature
T_d_: dew point temperature
T_g_: globe temperature
T_n_: natural wet-bulb temperature
USA: United States of America
W: wind speed
WBGT: wet-bulb globe temperature
ECMWF: European Centre for Medium-Range Weather Forecasts
VIF: variance inflation factor
